# Bleomycin‐induced flagellate hyperpigmentation

**DOI:** 10.1002/ccr3.831

**Published:** 2017-02-24

**Authors:** Krista N. Larson, Amy L. Gagnon, Barbara B. Wilson

**Affiliations:** ^1^ Department of DermatologyUniversity of Virginia School of MedicineCharlottesvilleVirginiaUSA

**Keywords:** Bleomycin, Hodgkin's lymphoma, hyperpigmentation

## Abstract

A 27‐year old male with Hodgkin's lymphoma was treated with combined chemotherapy that included bleomycin. He presented with pruritic erythematous, edematous linear lesions and was diagnosed to have flagellate hyperpigmentation, a rare side effect of bleomycin chemotherapy.

## Case Report

A 27‐year old male with a past medical history of Hodgkin's disease on a chemotherapeutic regimen including doxorubicin, bleomycin, vinblastine, and dacarbazine presented with a rash on his body that appeared after his first chemotherapy session 4 weeks earlier and worsened after the second chemotherapy session 2 weeks later. The rash began on his arms and spread to his back and forehead. The rash was pruritic, and the patient tried using 1% hydrocortisone cream with no improvement. He did note some relief of itching following a short course of prednisone. The patient has no personal history of skin disease and denied ingestion of shiitake mushrooms.

**Figure 1:**
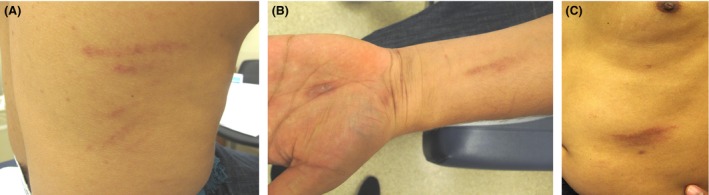
Clinical photographs of erythematous, edematous flagellate hyperpigmentation due to bleomycin on the (A) flank, (B) dorsal left arm and hand, and (C) trunk.

On physical exam, there were erythematous‐brown, edematous, linear dermal plaques on the trunk, extremities, left forehead, and abdomen (Figure. 1). There was an eczematous appearing red‐brown plaque on the left forearm. The differential diagnosis included contact dermatitis due to poison ivy, flagellate mushroom dermatitis, flagellate dermatitis secondary to dermatomyositis, or flagellate dermatitis secondary to adult‐onset Still's disease 1. As this patient did not consume shiitake mushrooms and did not have symptoms associated with dermatomyositis or adult‐onset Still's disease, but did have a history of bleomycin chemotherapy, a diagnosis of flagellate hyperpigmentation secondary to bleomycin was made.

Flagellate hyperpigmentation secondary to bleomycin is a rare side effect. Toxic levels of bleomycin accumulate in the skin as well as the lungs due to a low concentration of bleomycin hydrolase in these areas 2. There are many hypotheses for how the flagellate hyperpigmentation develops, including a localized increase in melanogenesis, toxic effects of the drug itself or localized drug eruption secondary to its leakage from blood vessels after scratching by the patient, and alterations to normal pigmentation patterns secondary to inflammation 3. Most often, the linear hyperpigmentation and erythema are preceded by a prodrome of pruritus which leads to the scratching by the patient 4. Patients typically start with erythematous, edematous lesions that later fade over months into the typical hyperpigmented lesions 5.

Many case reports of flagellate hyperpigmentation due to bleomycin have been published secondary to treatment with bleomycin for Hodgkin's lymphoma and other cancers 3, 6, 7. Flagellate hyperpigmentation has also been documented secondary to intake of shiitake mushrooms, adult‐onset Still's disease, and dermatomyositis 6. In addition to causing flagellate hyperpigmentation in the skin, bleomycin has been reported to cause Raynaud's phenomena, gangrene, fibrosis, neutrophilic eccrine hidradentitis (NEH), alopecia, edema, and nail changes 4.

Our patient was treated with daily antihistamines (cetirizine 10 mg) for the pruritus and topical clobetasol cream. In summary, although a rare side effect of bleomycin chemotherapy, flagellate hyperpigmentation should be considered in patients presenting with a pruritic linear rash.

## Authorship

KNL: Wrote the majority of the original draft, participated in generating/gathering data for the study, and approved the final draft of the paper. ALG: Participated in writing the paper and approved of the final draft of the paper. BBW: Participated in writing the paper and approved of the final draft of the paper.

## Conflict of Interest

None declared.
